# Plasma Acylcarnitines and Amino Acid Levels As an Early Complex Biomarker of Propensity to High-Fat Diet-Induced Obesity in Mice

**DOI:** 10.1371/journal.pone.0155776

**Published:** 2016-05-16

**Authors:** Olga Horakova, Jana Hansikova, Kristina Bardova, Alzbeta Gardlo, Martina Rombaldova, Ondrej Kuda, Martin Rossmeisl, Jan Kopecky

**Affiliations:** 1 Department of Adipose Tissue Biology, Institute of Physiology of the Czech Academy of Sciences, Prague, Czech Republic; 2 Department of Mathematical Analysis and Applications of Mathematics, Faculty of Science, Palacky University in Olomouc, Olomouc, Czech Republic; GDC, GERMANY

## Abstract

Obesity is associated with insulin resistance and impaired glucose tolerance, which represent characteristic features of the metabolic syndrome. Development of obesity is also linked to changes in fatty acid and amino acid metabolism observed in animal models of obesity as well as in humans. The aim of this study was to explore whether plasma metabolome, namely the levels of various acylcarnitines and amino acids, could serve as a biomarker of propensity to obesity and impaired glucose metabolism. Taking advantage of a high phenotypic variation in diet-induced obesity in C57BL/6J mice, 12-week-old male and female mice (*n* = 155) were fed a high-fat diet (lipids ~32 wt%) for a period of 10 weeks, while body weight gain (**BWG**) and changes in insulin sensitivity (**ΔHOMA-IR**) were assessed. Plasma samples were collected before (week 4) and after (week 22) high-fat feeding. Both univariate and multivariate statistical analyses were then used to examine the relationships between plasma metabolome and selected phenotypes including BWG and ΔHOMA-IR. Partial least squares-discrimination analysis was able to distinguish between animals selected either for their low or high BWG (or ΔHOMA-IR) in male but not female mice. Among the metabolites that differentiated male mice with low and high BWG, and which also belonged to the major discriminating metabolites when analyzed in plasma collected before and after high-fat feeding, were amino acids Tyr and Orn, as well as acylcarnitines C16-DC and C18:1-OH. In general, the separation of groups selected for their low or high ΔHOMA-IR was less evident and the outcomes of a corresponding multivariate analysis were much weaker than in case of BWG. Thus, our results document that plasma acylcarnitines and amino acids could serve as a gender-specific complex biomarker of propensity to obesity, however with a limited predictive value in case of the associated impairment of insulin sensitivity.

## Introduction

It is well established that environmental factors such as the lack of physical exercise and chronic nutrient overload combine with the susceptibility genes to induce obesity as well as an array of associated metabolic disorders including insulin resistance, hypertension, dyslipidemia, and impaired fasting glucose or glucose intolerance. This cluster of chronic diseases, i.e. metabolic syndrome, represents a risk factor for the development of type 2 diabetes and cardiovascular disease, thus being the major health threat in the developed countries [1]. The importance of the environmental factors in inducing these diseases is exemplified by the studies of healthy monozygotic twins who were discordant for obesity [2, 3]. For instance, it has been shown that obesity, independent of genetic influences, was primarily related to deleterious alterations in the spectrum of various lipid molecular species [2] and increased serum levels of insulin secretion-enhancing branched-chain amino acids (**BCAA**; [3]), which were associated with insulin resistance. Apparently, high throughput metabolomics analyses of serum/plasma samples using liquid chromatography coupled to mass spectrometry are useful for the identification of metabolites that represent a direct readout of biological processes, which are associated with cardiovascular and metabolic diseases ([2, 4, 5], and reviewed in [6]). At the same time, these approaches might be also used for the identification of biomarkers or a set of biomarkers that, besides being utilized for diagnosis or monitoring of disease, will be also predictive of incident disease [7, 8].

With regard to various components of the metabolic syndrome, high-fat feeding in rats [9, 10] or mice [4, 11, 12] is frequently used to induce obesity as well as insulin resistance, dyslipidemia and glucose intolerance. In these animal models of obesity, a number of metabolomics approaches has been already used to successfully identify metabolites such as various acylcarnitines (**AC**) or BCAA, which might be linked to developing insulin resistance in obesity (see refs. [4, 13, 14] and also reviewed in [8]). Interestingly, however, it was observed in genetically homogenous C57BL/6J (**B6**) mice [15, 16], that after a long period (~9 mo) of high-fat feeding the development of obesity and glucose intolerance was not uniform, and groups of lean and obese mice with either diabetic or nondiabetic phenotype can be distinguished. It has been suggested [16] that this differentiated metabolic response to the nutritional stress is due to epigenetic mechanisms influencing gene expression patterns and metabolic fates in each individual. B6 mice thus represent a molecular model to investigate non-genetic mechanisms of obesity. Koza et al. [17] documented that the phenotypes of male B6 mice characteristic of high or low gainers were already evident by 6 weeks of age, when mice were still on a low-fat diet.At the same time, human [18, 19] as well as animal [20–23] studies demonstrate less severe obesity-related metabolic disorders and/or later onset of these adverse phenotypes in female than in male subject. The fact that estrogen-deficient female mice exhibited an increased visceral fat mass as well as the expression of lipogenic genes [24], and that ovariectomized females had a higher propensity for the development of liver steatosis and insulin resistance [25], suggests that some estrogen-related mechanisms underlie the relatively low susceptibility of female mice to diseases related to the metabolic syndrome under conditions of high-fat feeding.

Biomarkers predicting obesity and associated disorders would be useful in order to enable appropriate lifestyle changes preventing the onset of such disease. However, reliable biomarkers are still not available. In this study, taking advantage of our established model of obesity induced by high-fat feeding [26–33], we aimed to identify potential biomarkers of propensity to obesity and insulin resistance using large cohorts of both male and female B6 mice as well as a high-throughput mass spectrometry-based screening of plasma metabolites, primarily AC and amino acids, analyzed before and after 10 weeks of high-fat feeding. Our results suggest that plasma AC and amino acids could serve as a gender-specific complex biomarker of propensity to obesity, however with a limited predictive value in case of the associated impairment of insulin sensitivity.

## Materials and Methods

### Animals and experimental setup

**Fig 1:**
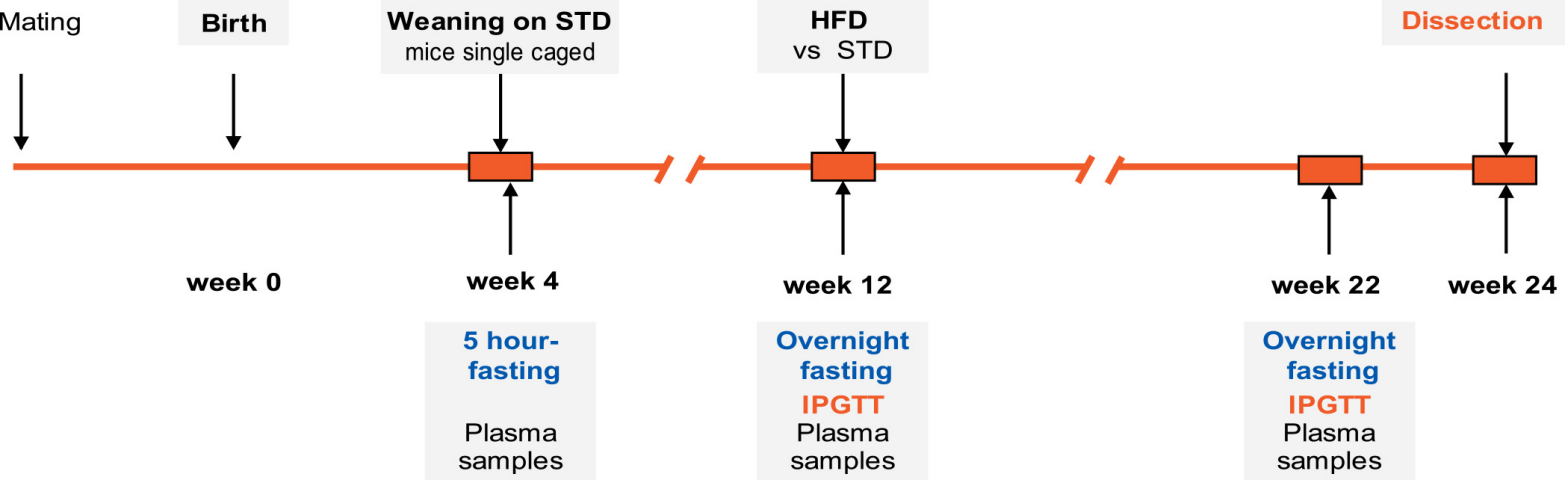
An overview of the experimental setup. Mice were weaned at the age of 4 weeks and fed a standard laboratory chow (STD) until 12 weeks of age, when they began to be fed a high-fat diet (HFD) until the end of study at the age of 24 weeks. Intraperitoneal glucose tolerance test (IPGTT) was performed before (week 12) and towards the end of high-fat feeding (week 22).

Mice of the C57BL/6J genetic background (from the colony maintained at the Institute of Physiology of the Czech Academy of Sciences, Prague) were kept in a controlled environment, i.e. at 22°C, 50% humidity, and 12h/12h light/dark cycle, with drinking water and diet *ad libitum*. Mice were fed a standard maintenance diet (**STD**; Ssniff R/M-H diet, Ssniff Spezialdieten GmbH, Soest, Germany), containing 13.0 kJ/g as proteins (33%), carbohydrates (58%), and lipids 9%). During mating at the age of 3–6 months, one male and one virgin female were kept together for 7–10 days. During pregnancy and after giving birth, female mice and subsequently also their pups were fed until weaning a standard breeding diet (Ssniff M-Z diet, Ssniff Spezialdieten GmbH, Soest, Germany), containing 13.9 kJ/g as proteins (36%), carbohydrates (53%), and lipids (11%). Three days after birth, the number of pups in each nest was reduced to 4–6. At weaning at the age of 4 weeks, mice were single-caged and fed the STD diet (see above) until 12 weeks of age. Thereafter, mice were randomly assigned to a corn oil-based high-fat diet (**HFD**; lipids ~35% wt/wt; see also ref. [26]) or remained on the STD diet as lean controls. Food intake and body weight was recorded weekly. In total, 73 females and 82 males in the HFD group and 11 females and 14 males in the STD group were analyzed. EDTA-plasma was obtained from tail blood collected at week 4, 12, and 22, and stored at -80°C for further analyses. Animals in ad libitum fed state were killed by cervical dislocation under diethylether anesthesia at week 24 (see **Fig 1**for the overview of experimental setup). All animal experiments were approved by the Animal Care and Use Committee of the Institute of Physiology, Czech Academy of Sciences (Approval Number: 172/2009) and followed the guidelines.

### Glucose homeostasis

Intraperitoneal glucose tolerance tests (**IPGTT**) were used to analyze glucose tolerance in mice after an overnight fast (6:00 p.m.– 9:00 a.m.) either before (week 12) or following a high-fat feeding (week 22). Blood glucose (glycemia) was measured using glucometers OneTouch Ultra (LifeScan, Milpitas, CA, USA) in samples of tail blood collected immediately before (0 min; i.e. in the fasted state), and 15, 30, 60, 120, and 180 min after the injection of D-glucose (1 g per kg body wt.) as before [28]. The level of glucose tolerance was determined as an incremental area under the glucose curve (**AUC**). Moreover, blood glucose levels in the fasted state (0 min) as well as their increase during the first 30 min of IPGTT (**ΔGlucose**) were used as additional indicators of the state of glucose homeostasis; a similar approach was used to calculate the change in plasma insulin levels (**ΔInsulin**). The homeostasis model assessment was used to quantify the index of insulin resistance (**HOMA-IR**) using the following formula: fasting plasma insulin (uU/ml) x fasting plasma glucose (mg/dl)/405.

### Plasma metabolites and hormones

Plasma concentrations of triacylglycerols and non-esterified fatty acids (**NEFA**) were measured using a diagnostic kit Triacylglycerols liquid 250 S (Erba Lachema, Brno, Czech Republic) and NEFA C kit (Wako Chemicals, Richmond, VA), respectively. For the measurement of insulin levels, EDTA-plasma was isolated from tail blood collected at the fasted state as well as 30 minutes after the glucose injection during IPGTT, and immunoreactive insulin was detected by a Mouse ultrasensitive Insulin ELISA kit (ALPCO, Salem, NH).

### Metabolomic analysis

The levels of various AC and amino acids in plasma were quantified by using a FIA-ESI-MS/MS and MassChrom® Amino Acids and Acylcarnitines kit (Chromsystems, Gräfelfing Germany), which represents an expanded screening panel of inborn metabolic disorders based on tandem mass spectrometry (see also refs. [13, 14]). Plasma samples for metabolomic analysis were collected from 5-h fasted mice at week 4 (weaning), and from overnight fasted mice at week 22 (i.e. after 10 weeks of high-fat feeding; see **Fig 1**). The concentration of carnitine (**Car**), short- (C2-7), medium- (C8-14), and long-chain (C16-20) AC, as well as of amino acids was determined in 1μl-aliquots of plasma using internal deuterated standards.

### Statistical analysis

Data are presented as means±SD. Comparisons were judged to be significant at *p*≤0.05. Data were processed as compositional data [34] in R software [35] with standard statistical packages and a specialized package robCompositions [36]. Zero imputation was applied on the dataset. These were imputed by two-thirds of the minimum for the appropriate analyte. Data were transformed with clr transformation [34, 37] and mean centered. The statistical evaluation was done through unsupervised and supervised methods, namely Principal Component Analysis (**PCA**) and Partial Least Squares-Discriminant Analysis (**PLS-DA**), respectively. The ellipses with 75% quantile were displayed in all score graphs. The evaluation of the PLS-DA was done with variable influence on projection (**VIP**) score [38]. Analytes with VIP > 1 were evaluated as significant. The normality test evaluated non-normal distribution of the data; because of this fact, the nonparametric Wilcoxon and Kruskal-Wallis tests were also used for the statistical analysis. Spearman’s correlations were calculated for pairs using SigmaStat 3.5 statistical software and considered as significant when p value was lower than 0.05 (Systat Software, CA, USA).

## Results

### Development of obesity and associated metabolic impairments induced by high-fat feeding

**Fig 2:**
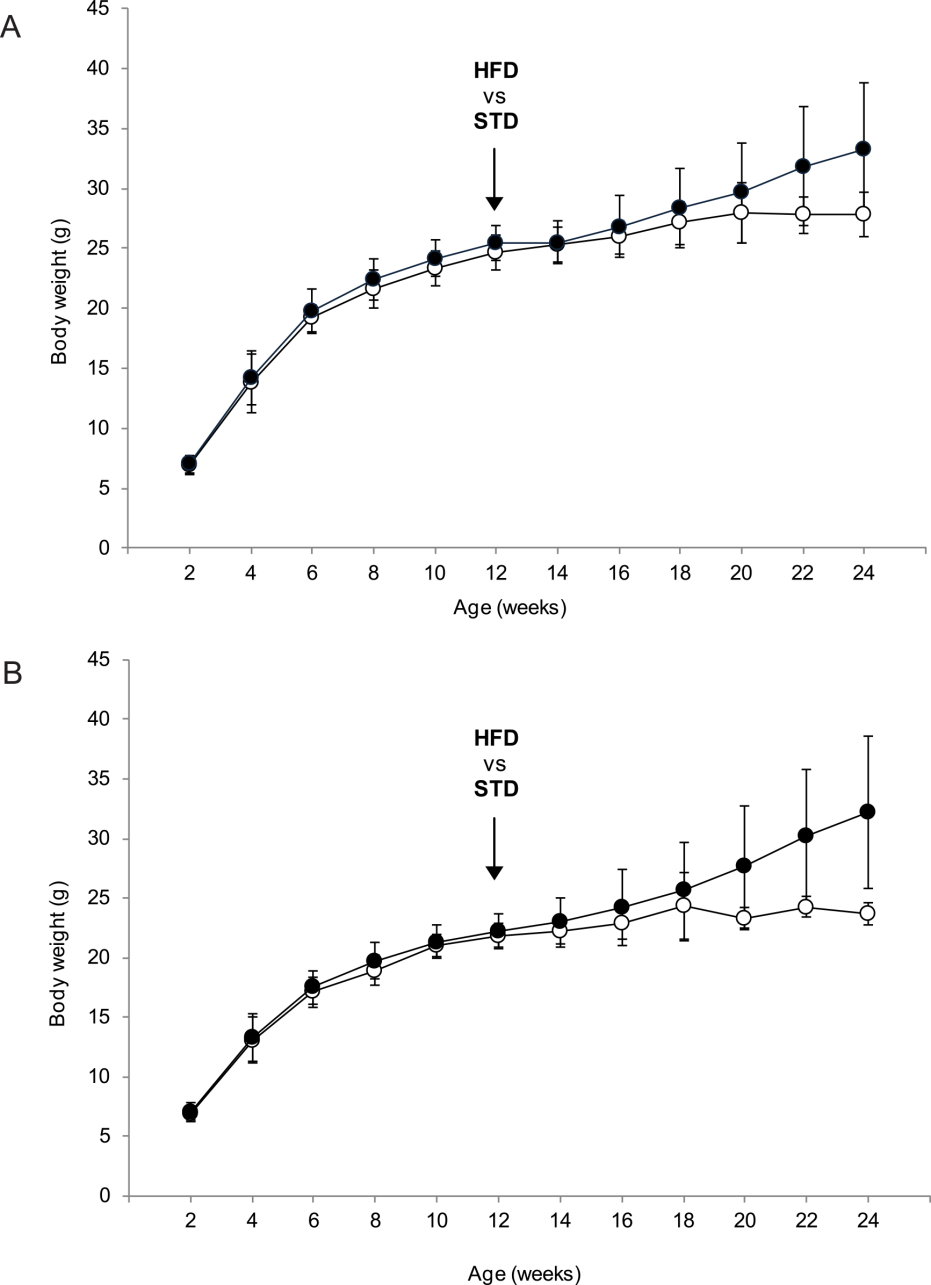
Body weight of mice before and during the development of obesity. Body weight of mice was monitored at the 2-week intervals starting at 2 weeks of age and continuing until the end of experiment (week 24). All mice were fed a standard laboratory chow (STD) until the age of 12 weeks, when most of the mice received a high-fat diet (HFD); the remaining mice continued on the STD diet until the end of study. Both male (**A**) and female (**B**) mice were followed during the experiment. The data are means ± SD (STD, empty circles, *n* = 14 (male) or 11 (female); HFD, black circles, *n* = 82 (male) or 73 (female)).

**Fig 3:**
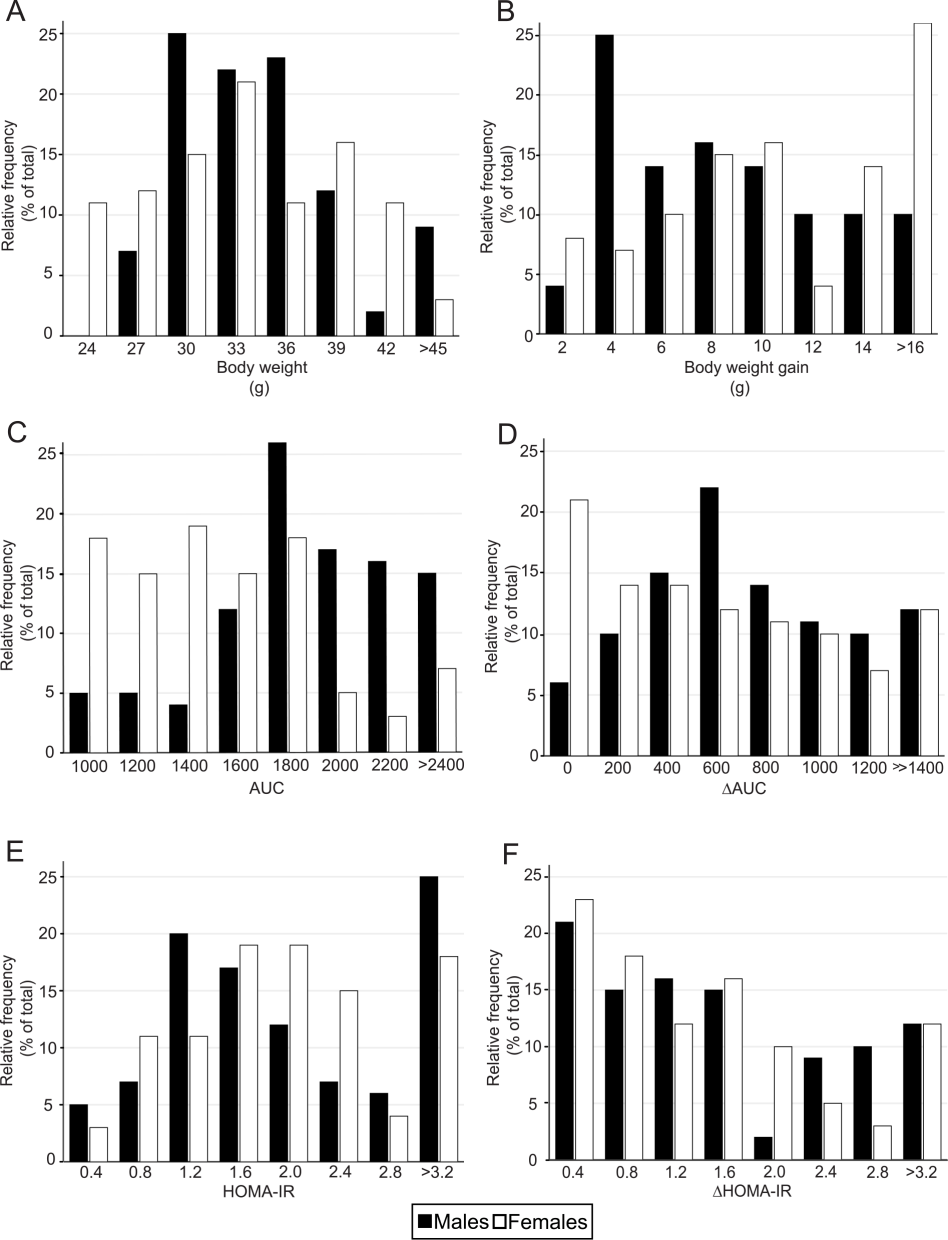
The distribution of selected obesity-associated phenotypes within the groups of HFD mice. The following parameters including body weight at week 24 (**A**) as well as body weight gained between the week 12 and 24 of the experiment (**B**), glucose tolerance assessed as the area under the glucose curve (AUC) at week 22 (**C**) as well as its change (ΔAUC) between the week 12 and 22 (**D**), and insulin resistance assessed as HOMA-IR at week 22 (**E**) as well as its change (ΔHOMA-IR) between week 12 and 22 (**F**) were assessed and their intra-group distributions were plotted. Both male (black bars; *n* = 82) and female (white bars; *n* = 73) mice were analyzed.

In total, 84 female and 96 male B6 mice were monitored with regard to their body weight and body weight gain (**BWG**) from 2 weeks of age until the end of study at week 24 (see **Fig 1**for the overview of experimental setup). Although females had a lower body weight than males from weaning until week 12, following the start of high-fat feeding their body weight increased more rapidly and was more heterogeneous (**Fig 2**and **Table 1**). Thus, at the end of experiment the range of body weight was 25–55 g in HFD males and 21–59 g in HFD females (**Fig 3A**), which was also reflected by a greater and more scattered BWG in the latter group (**Fig 3B**). Furthermore, HFD female mice as a whole (*n* = 73) showed significantly increased body weight after 4 weeks of high-fat feeding when compared with their STD counterparts, while in HFD males (*n* = 82) this difference was apparent only after 8 weeks of high-fat feeding (data not shown).

**Table 1:** Body weight and metabolic phenotypes of mice before and after high-fat feeding.

		Males		Females	
		STD	HFD	STD	HFD
***n***		14	82	11	73
**Body weight (g)**					
Week 12		24.7±1.4	25.5±1.5^c^	21.6±0.9^a^	22.2±1.4
Week 24		27.9±1.9	33.3±5.5^c^	23.7±0.9^a^	32.2±6.4^b^
BWG		3.2±1.0	7.8±4.9^a^^c^	2.2±0.9^a^	10.0±5.7^b^
**Glucose basal (mg/dl)**					
Week 12		104±14	107±20	119±23	111±21
Week 22		111±35	118±26^c^	95±22	106±23
**∆**		7±34	11±35^c^	-23±17	-6±31
**∆Glucose (mg/dl)**					
Week 12		257±49	236±47^c^	198±60^a^	209±69
Week 22		223±81	274±69^a^	209±63	277±68^b^
AUC glucose (mmol * 180 min)					
Week 12		1304±288	1160±290^a^	876±262^a^	955±350
Week 22		1341±517	1774±420^a^^c^	1148±332	1433±512^b^
**∆**		130±757	642±497^a^	271±363	504±634
**Insulin (μU/ml)**					
Week 12		1.7±1.5	2.6±1.9	1.7±1.3	2.7±2.4
Week 22		4.5±4.1	7.9±8.2^a^	3.4±2.2	7.7±4.5^b^
**∆**		2.9±4.6	5.4±8.1	1.9±2.5	5.1±5.0^b^
**∆Insulin (μU/ml)**					
Week 12		6.9±4.2	7.9±4.4	5.6±2.1	7.3±4.0
Week 22		6.9±7.0	9.4±7.4	7.8±5.9	9.1±5.9
**HOMA Index**					
Week 12		0.4±0.4	0.7±0.5	0.5±0.4	0.7±0.6
Week 22		1.4±1.9	2.5±3.3^a^	0.8±0.5	2.1±1.5^b^
**∆**		1.0±1.9	1.8±3.3	0.4±0.6	1.4±1.7^b^
**NEFA (mmol/l)**					
Week 12		0.6±0.1	0.6±0.2	0.5±0.1	0.6±0.3
Week 22		0.5±0.2	0.4±0.1^a^	0.5±0.2	0.4±0.1^b^
**Triacylglycerols (mmol/l)**					
Week 12		0.4±0.1	0.4±0.2	0.4±0.2	0.4±0.2
Week 22		0.3±0.1	0.4±0.1^a^	0.3±0.1	0.4±0.1^b^

Body weight and metabolic phenotypes were assessed in male (M) and female (F) mice fed either a standard chow (STD) or high-fat (HFD) diet. High-fat feeding was initiated at 12 weeks of age, and continued for the subsequent 12 weeks (week 24); metabolic parameters were assessed at week 22, i.e. after 10 weeks of high-fat feeding. Blood glucose and plasma insulin have been measured after an overnight fast, right before the injection of glucose to assess glucose tolerance. Data are means ± SD. AUC, area under the glucose curve; BWG, body weight gain; NEFA, non-esterified fatty acids; ∆, difference between the values of a given parameter measured at week 22 and week 12; ∆Glucose and ∆Insulin, the change in blood glucose and plasma insulin levels, respectively, assessed before and 30 min after the injection of glucose in the context of glucose tolerance testing.

^a^significantly different from Males STD

^b^significantly different from Females STD

^c^significantly different from Females HFD.

At week 12, just before the start of high-fat feeding, glucose tolerance was better (i.e., AUC was lower) in female than in male mice, while fasting blood glucose and plasma insulin levels were similar in both genders (**Table 1**). However, the value of ∆Glucose but not ∆Insulin (see Materials and Methods for the definition of these values) was higher in males when compared to females (**Table 1**). At week 22, after high-fat feeding, glucose tolerance was impaired in HFD mice of both genders (as compared to their STD counterparts), however significantly more in HFD males; the ∆Glucose value increased to a similar extent in male and female HFD mice (**Table 1**). In analogy to variations in BWG (see above), also the overall level of glucose tolerance at week 22 and its change from basal values (i.e. at week 12) showed substantial differences among individual HFD mice of both genders (see **Fig 3C and 3D**). Although fasting blood glucose levels were higher in male than in female HFD mice, they were not significantly increased when compared to their STD counterparts; in contrast, fasting plasma insulin was elevated to a similar degree in HFD mice of both genders (**Table 1**). As expected, the HOMA-IR index was also increased (**Table 1**), suggesting an impairment of insulin sensitivity in both male and female HFD mice, however with large interindividual differences observed for both the HOMA-IR index and its change from basal values (see ΔHOMA-IR in **Table 1**and **Fig 3E and 3F**). In contrast to various parameters of glucose homeostasis, plasma levels of lipid metabolites including NEFA and triacylglycerols measured in HFD mice at week 22 showed little changes when compared to their respective STD counterparts (**Table 1**).

**Fig 4:**
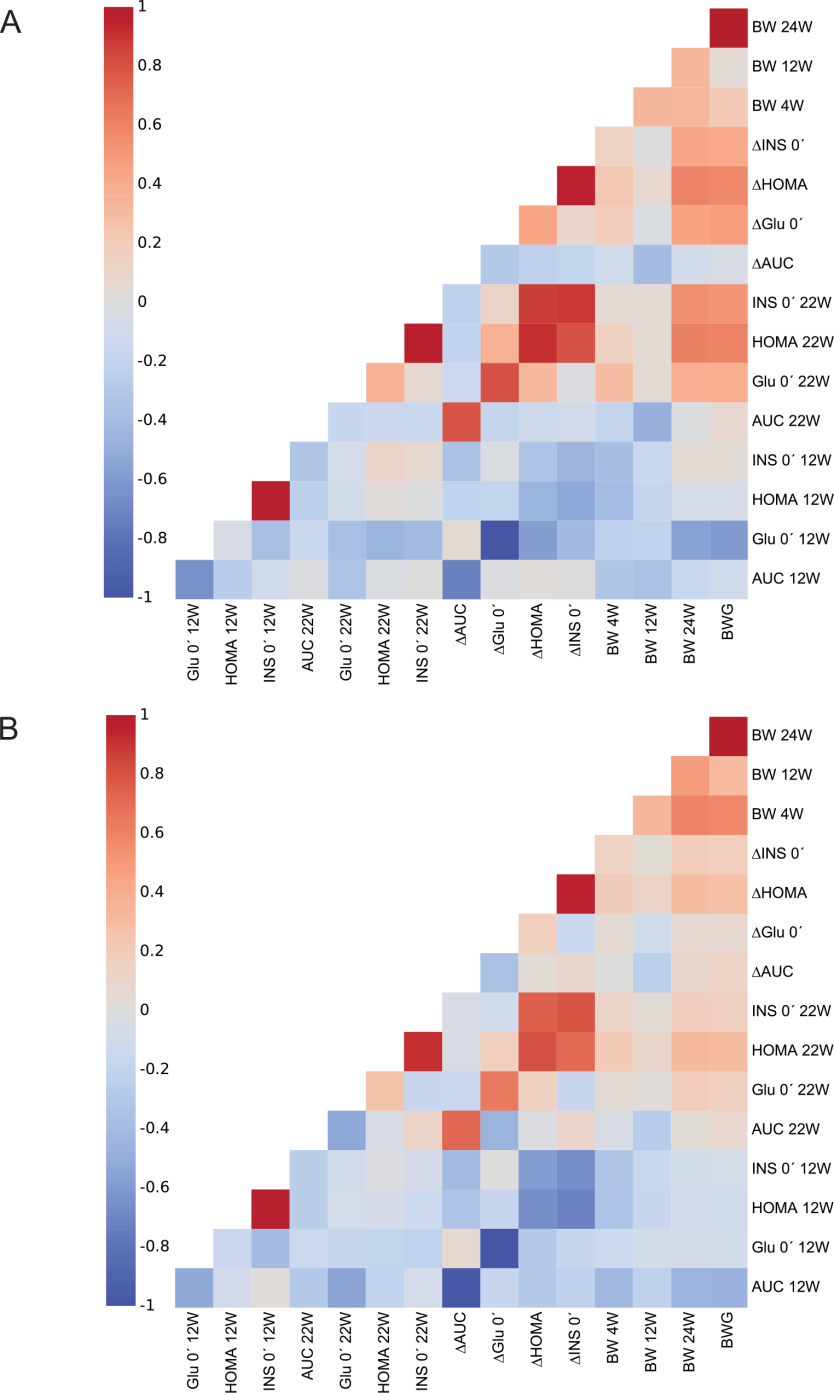
Correlation analysis of various metabolic parameters assessed before and after high-fat feeding. Before the induction of obesity by a high-fat diet (week 12–24), mice were fed a standard laboratory chow from weaning (week 4) until week 12. Spearman's rank correlation coefficients were generated to characterize the relationships between different variables associated with the weight gain and glucose homeostasis in both male (**A**) and female (**B**) mice. The data are presented as heatmaps, where different colors correspond to coefficient values of the respective correlations.

In order to characterize the interrelationship among various parameters of glucose homeostasis, as well as their relationship to body weight, the correlation analyses were performed (**Fig 4**). The parameters including AUC, HOMA-IR, fasting plasma insulin and blood glucose, assessed before the start of dietary intervention at week 12, all exhibited strong negative correlations with their respective Δ values (i.e. the change induced by high-fat feeding during the week 12–22), especially in female mice (**Fig 4**). However, these strong negative correlations could reflect the phenomenon called the regression to the mean (RTM), which is also supported by the fact that the correlation coefficients, describing the relationship between parameters of glucose homeostasis measured at week 12 and 22, are relatively weak (**Fig 4**). Despite the fact that the association between body weight assessed at week 4 (weaning) and week 24 (the end of study) was much stronger in females than in males (females, r_s_ = 0.68 vs. males, r_s_ = 0.44), the relationships (positive) between BWG and ΔHOMA-IR, ΔInsulin or ΔGlucose were much stronger in males than in females (**Fig 4**).

Thus, although male and female mice exhibit similar relationships among various parameters associated either with body weight or glucose homeostasis, their interrelationship differs between the genders.

### Metabolomic analysis of plasma AC and amino acids before and after the development of obesity

In total, plasma levels of 27 AC and 11 amino acids in the fasting state were evaluated at week 4 (weaning), and at week 22, i.e. 10 weeks after the start of high-fat feeding. Except for C20, which was decreased, all other analyzed AC species were increased between week 4 and week 22 in plasma of HFD animals (**S1**and **S2 Tables**). Plasma AC levels in STD mice showed a similar profile, however, the increase in the levels of Car, C3, C4, C8, and C20 in female mice at week 22 was not significant (**S1**and **S2 Tables**). Regarding the effect of high-fat feeding on plasma AC levels analyzed at week 22, the levels of nine AC species were higher, namely Car, C2, C3, C4, C4-OH, C10:1, C18:2 and C16-DC, while the levels of seven AC species were lower, namely C4-DC, C14:1, C14, C16:1, C16, C16-OH and C20, in HFD as compared to STD mice of both genders. Gender-specific differences in response to HFD were observed; while C10 and C18 species were increased only in HFD males, C5, C14:2, and C16:1-OH increased and C18:1, C18:1-OH and C20:4 decreased only in HFD females. Moreover, the metabolite C18-OH was oppositely regulated in HFD male and female mice, showing an elevation in males and a decrease in females when compared to their respective STD controls (**S2 Table**).

The changes in plasma amino acid levels between week 4 and week 22 in STD mice followed the same pattern as observed in HFD groups, but in general were much weaker (**S1**and **S2 Tables**). Regarding the effect of high-fat feeding on plasma amino acid levels analyzed at week 22, the levels of two amino acids were higher, namely Tyr and Trp, and the levels of Pro and Leu+Ile were lower in HFD as compared to STD mice of both genders. Gender-specific effects of intervention with a high-fat diet were observed in case of Orn, the levels of which were increased only in females; in contrast, plasma levels of ketogenic amino acid Lys were increased only in HFD males (**S1**and **S2 Tables**).

These data suggest a complex response of plasma AC and amino acids to high-fat feeding, with some of the metabolites being regulated in a gender-dependent manner.

### Correlation analysis to investigate the relationship between body weight gain or insulin sensitivity and plasma levels of AC or amino acids

**Fig 5:**
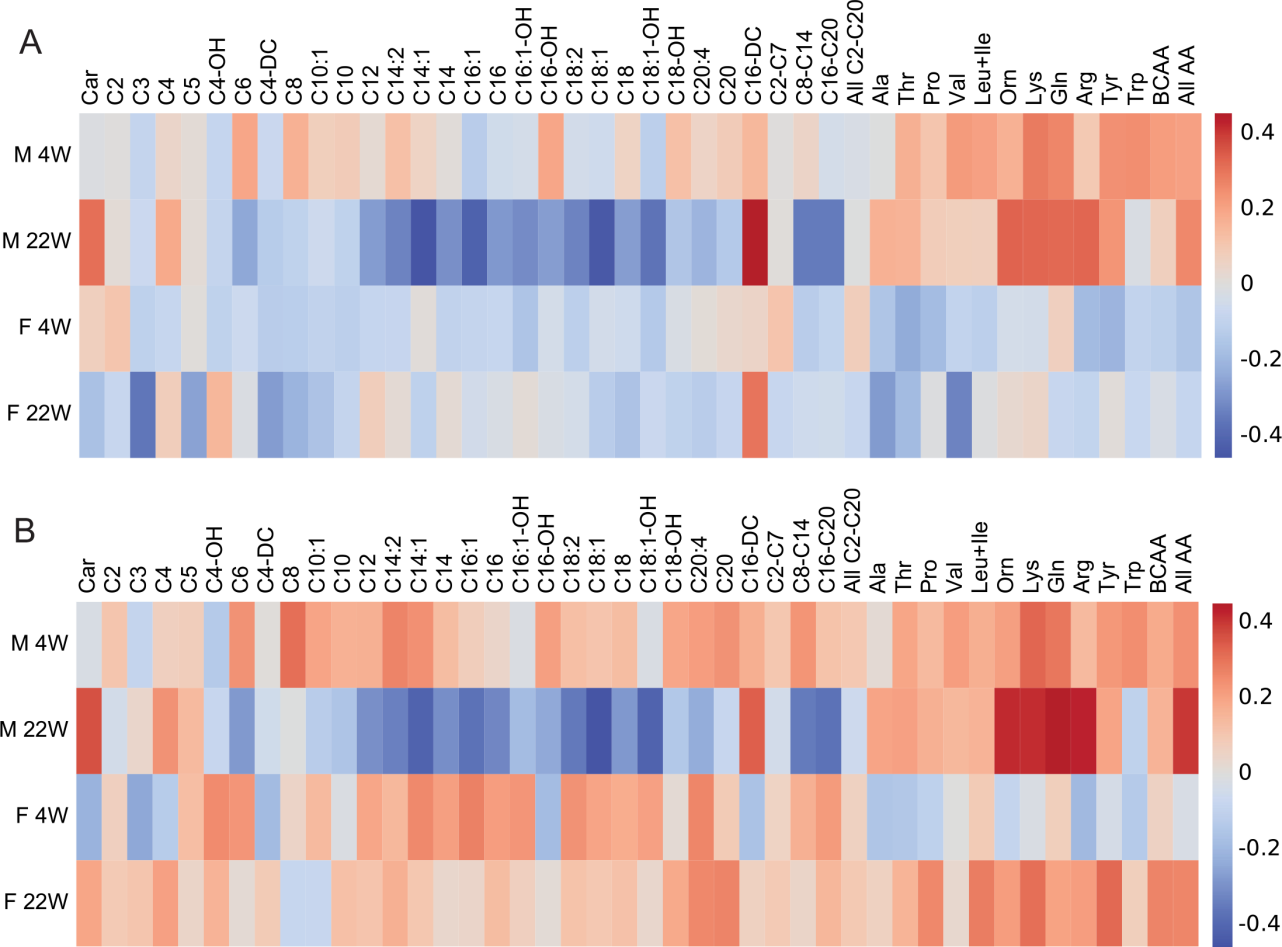
Correlation analysis of plasma AC species and amino acids with the degree of obesity and insulin resistance. Plasma levels of individual metabolites assessed in both male and female mice (M and F, respectively) at week 4 and 22 were correlated with the parameters of body weight gain during a 12-week period of high-fat feeding (i.e. week 12–24; **A**) and with the change in insulin sensitivity during a 10-week-period (i.e. week 12–22; **B**). Data are presented as heatmaps, where different colors correspond to coefficient values of the respective correlations (Spearman's rank correlation coefficients).

In order to reveal whether plasma AC and amino acids could serve as a marker of propensity to obesity and/or insulin resistance, univariate as well as multivariate data analyses were performed. First, Spearman's rank correlation coefficients were calculated to reveal the strength of association between the parameters such as BWG and ΔHOMA-IR and fasting plasma levels of individual AC or amino acids analyzed in HFD mice both before (week 4) and after (week 22) dietary intervention (**Fig 5**). Thus, at week 4, a number of significant but relatively weak (r_s_ < 0.3) correlations was found between BWG and amino acids but not AC, including a negative correlation with Thr in females and positive correlations with Val, Gln, Lys, Tyr, Trp and BCAA (Leu+Ile+Val) in males (**Fig 5A**). At week 22, plasma levels of Val, Ala, C3, C5 and C4-DC were negatively correlated with BWG in females, while in males negative correlations for C6, saturated (C12, C14, C16 and C18), unsaturated (C14:2, C14:1, C16:1, C18:2 and C18:1) and hydroxylated (C16-OH and C18:1-OH) long-chain AC species, and positive correlations for Car, Tyr, Orn, Gln, Lys and Arg were observed (**Fig 5A**). Only C16-DC showed a similar direction and strength of the correlation in both genders (female r_s_ = 0.30; male r_s_ = 0.45; p < 0.05).

Although in male mice the relationship between ΔHOMA-IR and various plasma AC and amino acid levels at week 4 or week 22 (**Fig 5B**) was very similar as in case of BWG (see above), in females at week 4 this relationship for most of the AC species except C3, C4, C4-DC, and C10 showed a different pattern (**Fig 5B**); in contrast, mostly negative correlations (except for Lys) observed between ΔHOMA-IR and amino acids were analogous to those with BWG. Interestingly, in females at week 22, except for C4-OH, C8, C10:1, and C12, the relationships between ΔHOMA-IR and either plasma AC or amino acids (**Fig 5B**) were completely opposite to those with BWG. Of note, the above associations of plasma AC and amino acids with either BWG (**Fig 5A**) or ΔHOMA-IR (**Fig 5B**) at week 22 were significant but relatively moderate (e.g. for BWG, C14:1, r_s_ = -0.46; C16:1, r_s_ = -0.42; C18:1, r_s_ = -0.44; for ΔHOMA-IR, C18:1, r_s_ = -0.43) or weak (r_s_ < 0.39). Thus, the similarities between the correlation patterns of either ΔHOMA-IR or BWG with various plasma AC and amino acids in males reflect a tight correlation between ΔHOMA-IR and BWG found only in these mice. However, although the correlation analyses of selected phenotypes with metabolomics data suggested potential biomarkers of propensity to obesity and/or insulin resistance, it was not possible to identify a strong candidate or candidates when using the entire mouse cohorts.

### Discriminant analysis of metabolomics data in selected groups of “low gainers”and “high gainers”according BWG and ΔHOMA-IR

In order to increase the likelihood of identifying plasma metabolites that can be used as biomarker(s) of obesity, we next used multivariate statistical analyses applied to a selected group of both HFD male and female mice, which comprised animals within the upper and lower 15% of the frequency distribution for BWG (week 12–24). The same approach was also used to select mice with either the lowest or highest change in insulin sensitivity based on the assessment of HOMA-IR (week 12–22; ΔHOMA-IR).

**Fig 6:**
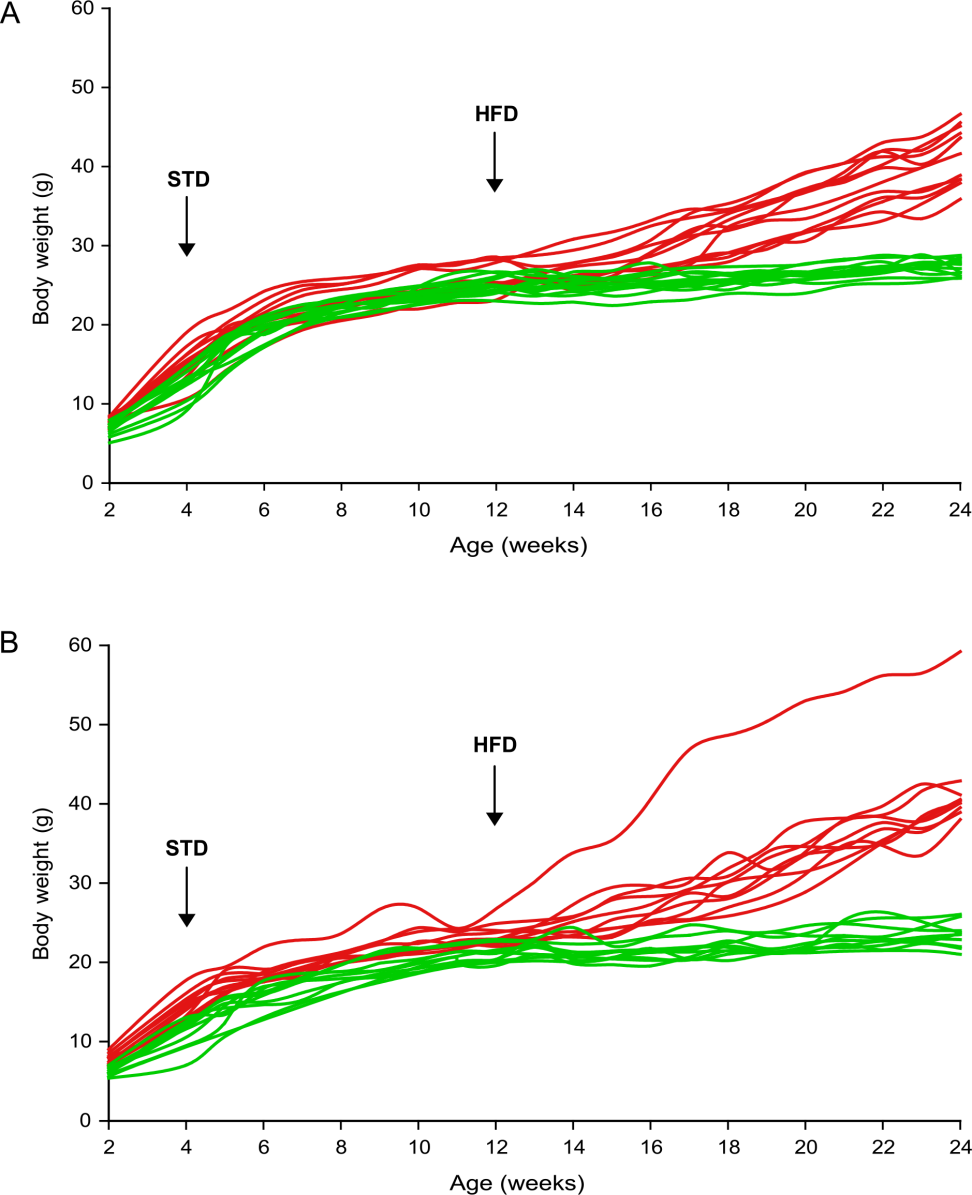
The course of changes in body weight in the selected groups of mice with the lowest and highest BWG. The animals within the upper and lower 15% of the frequency distribution for BWG were selected and their respective body weights were plotted for both male (**A**) and female (**B**) mice. Mice with the lowest (green color) and highest (red color) body weight gain for both genders are shown.

**Fig 7:**
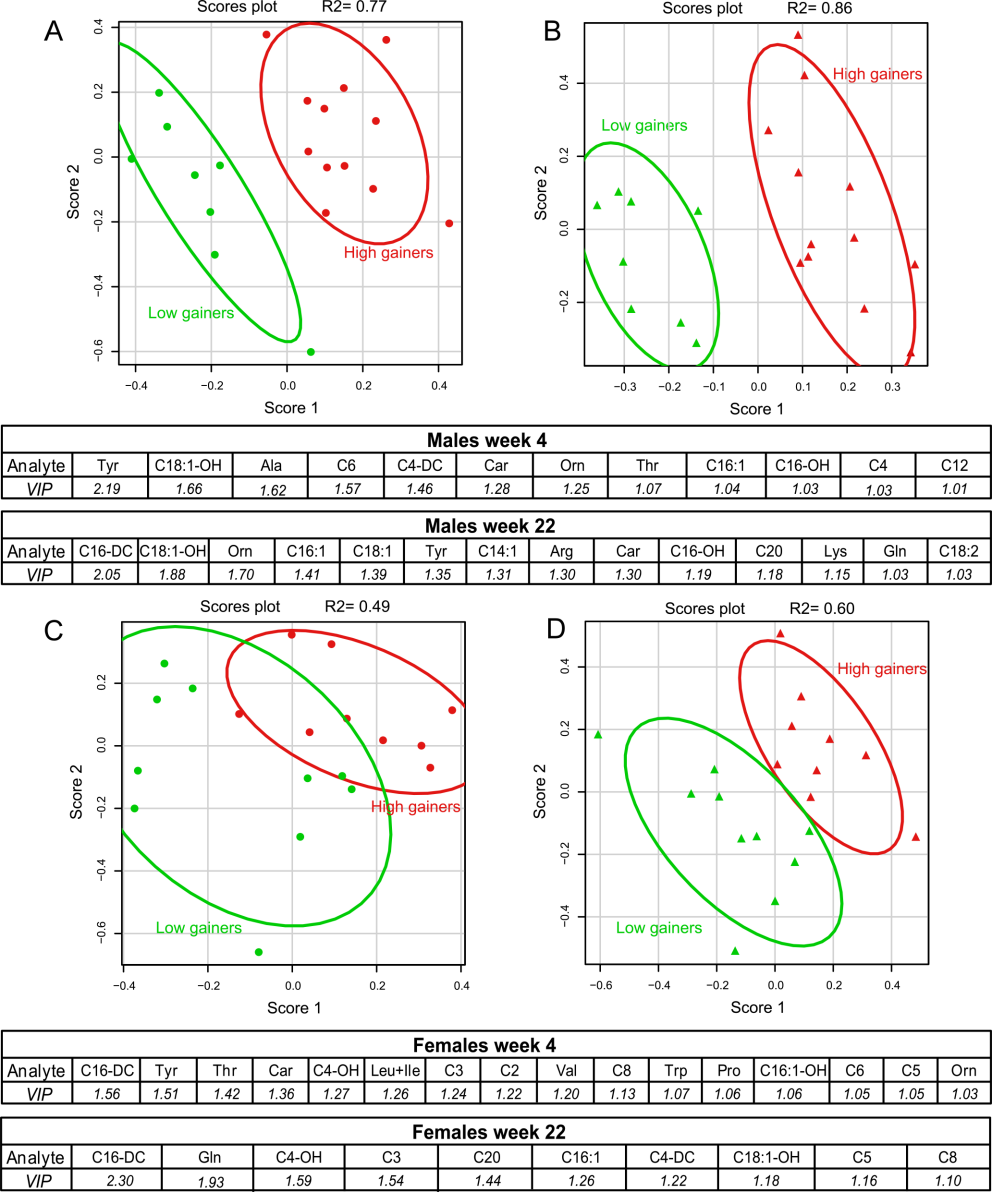
Multivariate analysis of plasma metabolome. Plasma levels of AC species and amino acids in the fasting state were analyzed by the PLS-DA analysis in groups of mice selected for the highest and lowest BWG. Score plots resulting from the PLS-DA analysis of plasma metabolite levels measured at week 4 (**A**, **C**) and week 22 (**B**, **D**) of the study were generated for both male (**A**, **B**) and female (**C**, **D**) mice. Plasma metabolites that were identified as those most discriminating between the groups of „high gainers”(red marks) vs. „low gainers”(green marks) in both genders are listed in the tables under the respective score plots; the metabolites are ranked according to their variable influence on projection (VIP) scores, and only those with VIP scores greater than 1 are shown. Three outliers based on PCA analysis and two animals due to missing values were excluded from further analysis.

In both male and female mice, body weight of animals selected for their low or high BWG (see above; “low gainers” and “high gainers”, respectively) tended to diverge already at week 4, with a stronger separation in female mice (**Fig 6**). By using the PLS-DA, a supervised classification method, to analyze plasma metabolome in males, a separation between the “low gainers” and “high gainers” into two distinct groups was obtained at week 4 as well as at week 22 (**Fig 7A and 7B**; R^2^ = 0.77 and 0.86, respectively). In female mice, the complete separation was obtained at week 22 (R^2^ = 0.60; **Fig 7D**), but, no clear separation was observed at week 4 (R^2^ = 0.49; **Fig 7C**). Furthermore, based on the VIP scores (see the section Statistical analysis in Materials and methods), a threshold of 1.0 was defined for the most discriminating metabolites distinguishing “low gainers”vs. “high gainers”(**Fig 7**; see the corresponding tables below the graphs); thus, among the most discriminating metabolites in male mice it was Car, C16:1, C16-OH, C18:1-OH, Tyr and Orn, which were present at both time points, i.e. at week 4 and week 22 (**Fig 7A and 7B**). While in male mice at week 4, Tyr was the only discriminating analyte showing a significant individual association with BWG (**Figs 5**and **7**), all discriminating metabolites at week 22 exhibited significant correlations with BWG (**see Figs 5**and **7**). In females, the most discriminating metabolites included C3, C5, C4-OH and C8 at both time points. Thr, Tyr, Orn, Car and C6 at week 4, as well as C16-DC, Gln, C16:1 and C18:1-OH at week 22, were the most discriminating metabolites irrespective of the gender (**Fig 7**; see the tables below the graphs).

**Fig 8:**
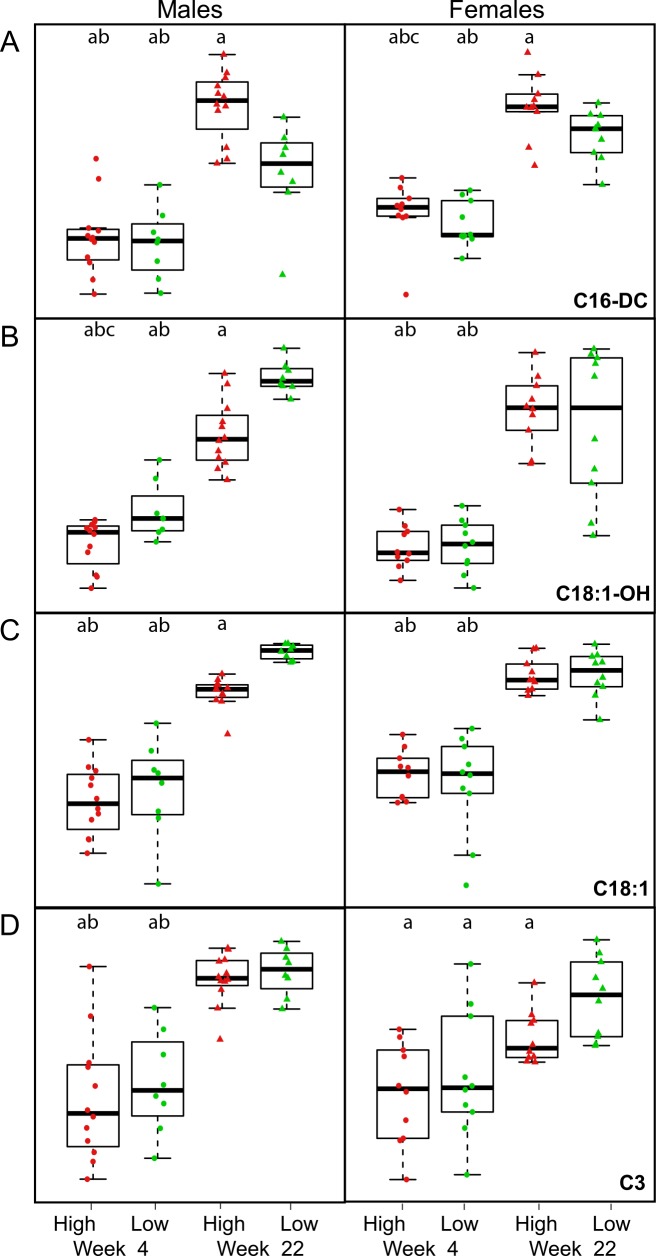
Plasma levels of selected metabolites showing a significant difference between the groups of mice with the lowest and highest BWG. Plasma levels of C16-DC (**A**), C18:1-OH (**B**), C18:1 (**C**), and C3 (**D**) were assessed at week 4 and week 22 (i.e. after 10 weeks of high-fat feeding) in groups of male and female mice showing the lowest (green marks) and highest (red marks) BWG. ^a^*p*<0.05 vs. „low gainers”at week 22; ^b^*p*<0.05 vs. „high gainers”at week 22; ^c^*p*<0.05 vs. „low gainers”at week 4.

The analysis of variance (**ANOVA**) of plasma levels of individual metabolites revealed, that in males at week 22 all the discriminating metabolites (except C20; see **Fig 7**and representative analyses on selected metabolites in **Fig 8**) and additionally also C12, C14:2, C14, C16:1-OH, Ala and Thr (not shown) differed between the groups of low and high gainers. Much less metabolites significantly differed either between low and high gainers in females at week 4 and 22, or in males at week 4, however all of them represented the discriminating metabolites identified by the PLS-DA (**Fig 7**); thus, in females, C16:1-OH and C16-DC differed at week 4 and C3, C4-DC and C16-DC at week 22, while in males C6, C4-DC, C18:1-OH and Tyr differed at week 4 (see the representative analyses on selected metabolites in **Fig 8**).

By contrast, the same approach utilizing PLS-DA as above and applied to the parameter of ΔHOMA-IR as a surrogate marker of insulin resistance (**S1 Fig**), revealed a less distinct separation between males with either low or high insulin resistance both at week 4 and 22 (**S1A and S1B Fig**; R^2^ = 0.55 and 0.69, respectively), and even weaker separations in females (**S1C and S1D Fig**; R^2^ = 0.44 and 0.44, respectively).

## Discussion

It is of utmost importance to identify a biomarker or a set of biomarkers that, besides being utilized for diagnosis or monitoring a particular disease, will be also predictive of incident disease [7, 8]. For identification of novel biomarkers, animal models are instrumental. With regard to obesity and its comorbidities, a large phenotypic variation is observed among various inbred strains of mice [39]. Further in this regard, variations within a highly inbred strain can also be used to uncover mechanisms that determine the obese phenotype while acting independently of inherited mutations to the genes; in other words, if mice of a given inbred strain are characterized by a stable but at the same time variable obese phenotype, it suggests the influence of epigenetic factors that act together with environment as well as with genetic factors [16, 17].

Thus, the aims of this study were: 1) to establish the variable obesity phenotype in a large cohort (*n* = 180) of inbred B6 mice of both sexes, i.e. the mouse strain that has been previously shown to develop variable degree of obesity when fed a high-fat diet [11, 15–17]; 2) to reveal presumed [20–23] gender-dependent differences regarding the development of obesity and associated metabolic disorders; and 3) to identify potential biomarkers of the propensity to obesity and/or insulin resistance in male and female B6 mice using metabolomics approach [5, 13, 14].

We show here that a chronic 12-week-long high-fat feeding has led to wide differences in body weight gain, which was documented by a maximum difference between the leanest and heaviest animal of ~30 g and ~38 g in male and female mice, respectively. Despite the fact that these large differences in weight gain do not represent a novel observation per se (for instance, see refs. [15, 17]), it served our attempts to identify metabolomics-based biomarkers underlying the propensity to obesity in mice of both genders. In order to minimize potential contributions of variations in food intake in the early postnatal period to differences in obesity development later in life [40–42], the size of litters was reduced to 4–6 mice shortly after birth. However, variations in litter size would probably be of minor importance and could contributed only ~3% to the variance in body adiposity [17]. Furthermore, in accordance with our previously published data [23], a more accelerated HFD-induced weight gain was observed in female as compared to male mice. Interestingly, the relative distribution of mice with the lowest and highest body weight at the end of study (week 24) was similar to that observed already at week 2 of their postnatal life. This „programming”effect has been previously observed in male B6 mice [17]; however, in our study it was female mice which showed a more distinct and persistent separation between mice with the lowest and highest body weight as well as tighter correlations between body weights assessed at week 4 (weaning) and 24 (dissection) (r_s_ = 0.69) when compared to also relatively strong correlation in male mice (r_s_ = 0.44).

As expected [11, 23], plasma triacylglycerol levels measured in overnight fasted animals at the end of experiment were increased in HFD mice as compared to STD controls, however, plasma NEFA levels were higher in STD mice. Such an observation is also complemented with a decrease in plasma levels of long-chain AC in HFD mice when compared to STD animals at week 22. One possible explanation could be that HFD mice did not respond properly to lipolytic stimuli that occur during fasting (as documented previously, see ref. [43]), resulting in an impaired NEFA release from adipose tissue.

In agreement with the previous reports [15, 16, 44], high-fat feeding in both male and female mice impaired insulin sensitivity (assessed as HOMA-IR) as compared to their STD-fed counterparts; however, the variations among individual mice regarding the degree of insulin resistance were substantial. Interestingly, the changes in the majority of parameters of glucose homeostasis (e.g. HOMA-IR, fasting plasma insulin, as well as the parameter of ∆Glucose–see Materials and Methods for the definition) were positively correlated with weight gain especially in males, while in female mice these correlations were not significant. Thus, other factors besides weight gain and/or increased adiposity, e.g. sex hormones (see ref. [45]), are likely to contribute to the regulation of insulin sensitivity in females. Given the fact that the samples in our study were collected over a period longer than one year, the influence of seasonal changes might be also considered. For instance, seasonal changes in plasma hormonal profiles [46] and pineal melatonin concentrations [47] have been previously reported among male laboratory animals with stable maintenance conditions. However, possible changes in plasma levels of other metabolites such as glucose or NEFA could also be involved.

The metabolomic analysis in our study was performed by utilizing the previously well-established screening method [5, 13, 14] based on the detection of various AC species and amino acids in plasma samples. While the assessment of plasma profiles of AC and amino acids is currently utilized for the early diagnosis of inherited disorders of fatty acid catabolism including medium and very long chain acyl-CoA dehydrogenase deficiencies, disorders of amino acid catabolism such as phenylketonuria and maple syrup urine disease, as well as organoacidurias (e.g. isovaleric acidemia) [48], the growing number of studies has provided evidence also about the involvement and/or influence of AC and amino acids with regard to development of insulin resistance ([3, 4, 49], and reviewed in [50]). Changes in the levels of AC and amino acids in plasma and tissues has been extensively studied not only in patients with insulin resistance and type 2 diabetes, but also in prediabetic patients [51]. Notably, BCAA and their metabolites C3, C5 and C4-DC have been implicated in the development of insulin resistance (refs. [3, 4] and reviewed in [8]). However, the knowledge regarding the alterations in AC and amino acid metabolism during development of obesity is still limited. For instance, long-chain AC species [52, 53] and the metabolite C4-OH [53] have been linked to the phenotypes of obesity and type 2 diabetes in humans.

Here, the metabolomic analysis was performed in the fasted state. It is known that plasma levels of long-chain AC species increase in response to fasting, thus reflecting higher rates of fatty acid oxidation [54]. Although the contribution of tissue metabolism to plasma AC levels remains unclear [55], it has been shown in pigs that liver could produce more long-chain AC species in the fasted state when compared to muscle and kidney [56]. Concurrently, using the same animal model, the rate of carnitine biosynthesis was shown to be increased in the liver and kidney in fasting [57]. On the contrary, insulin has been reported to reduce all plasma AC species in humans [53].

We first attempted to identify biomarker(s) of propensity to obesity in the whole cohorts of male and female HFD mice with their wide range of BWG. While only relatively weak correlations between BWG and some of the analyzed metabolites, especially amino acids, were observed in mice at week 4, i.e. before the onset of high-fat diet-induced obesity, several stronger correlations were found in obese mice at week 22; of note, moderate (r_s_ > 0.40) and negative correlations between BWG and even/long-chain AC species (e.g. C14:1, C16:1, C18:1, C18:1-OH) were noted particularly in male mice. A positive relationship between plasma long-chain AC species and body weight has been reported in humans when comparing obese and lean subjects [53]. The results of the human studies are in agreement with our finding that plasma levels of long-chain AC species are elevated in HFD animals when compared to STD mice (see **S2 Table**). Therefore, the diet could serve as a major contributor to the elevation of plasma levels of long-chain AC species in both mice and humans (see also ref. [58]).

A positive correlation observed in our study between BWG and Car in male HFD mice suggests a reduced activity of the enzyme carnitine palmitoyltransferase 1 (**CPT1**) in animals with a higher degree of obesity, which would then explain the negative correlation of BWG with plasma levels of even/long-chain AC species in these mice. This is also consistent with the findings in patients with a CPT1 deficiency [48], who have reduced levels of long-chain AC species with a simultaneous increase in free Car. Therefore, further analyses of the involvement of CPT1 with respect to the negative association between the degree of obesity and plasma levels of even/long-chain AC species is warranted.

In females at week 22, a particularly strong negative association was observed for Val, i.e. one of the BCAA, as well as for three AC species that are related to BCAA metabolism, namely C3, C5 and C4-DC. While these results are in contrast with the published data obtained in obese human patients [3, 4], another study in high-fat diet-fed mice has reported a decrease in serum BCAA levels in these obese animals [59]. Of note, the only exception to the gender-specific pattern of metabolite profiles in our study was the AC species C16-DC, which is related to peroxisomal fatty acid oxidation [60], and which exhibits a strong positive correlation in both male and female at week 22. This metabolite also exhibited a positive correlation with HOMA-IR at week 22, but only in male mice, which probably reflects the tight association of BWG with ΔHOMA-IR observed predominantly in mice of male gender.

In order to increase the likelihood of identifying a robust biomarker of propensity to obesity, we took advantage of a previously published approach [17] based on the selection of mice from the upper and lower extremes of frequency distribution for BWG coupled with multivariate analyses of metabolomics data. The PLS-DA type of analysis of metabolomics data (i.e. plasma levels of AC and amino acids in the fasting state) finally resulted in well-fitting models for obese and lean mice at week 4 and week 22 in case of males, but only for week 22 in females; in contrast, the PCA analysis was able to separate the groups of mice with low and high BWG only in case of males at week 22 (not shown).

In the context of obesity, several discriminating metabolites identified in our study have been also observed in human studies. In fact, the discrimination of the two groups with low and high BWG in males at week 22 was mostly based on long-chain AC species and selected amino acids, which represent the same metabolites that have been previously identified by the PCA analysis to be part of a complex biomarker distinguishing metabolically healthy vs. unhealthy phenotype within the mixed cohort of lean, overweight and obese subjects [61]. Moreover, tyrosine and glutamine together with phosphatidylcholines have been shown as metabolites important for prediction of visceral adiposity in healthy obese humans [62], while plasma levels of alanine, glycine, glutamate, tryptophan, tyrosine and BCAA were associated with visceral fat accumulation in obese Japanese subjects [63]. Mice with diet-induced obesity were characterized by increased plasma levels of ornithine in response to a high-fat diet administered for a period of 12 weeks [64].

In our study, long-chain AC species and Car belonged to the most discriminating factors already at week 4, i.e. in the pre-obese state, and thus together with even short AC species (C4, C6), BCAA-derived C4-DC and amino acids Tyr, Ala, Orn and Thr represent a complex, early biomarker of obesity in male B6 mice. In contrast, only a few long-chain AC species played a discriminating role in females with low and high BWG at the end of study, with a greater influence observed in case of BCAA and their metabolites both for BWG and ΔHOMA-IR; however, the low accuracy of all PLS-DA models in female mice indicates that changes in the metabolism of fatty acids, BCAA and ketone bodies, represented by plasma AC and amino acid levels, do not represent the main processes underlying the development of obesity and insulin resistance in this gender.

In conclusion, our study in a large cohort of B6 mice that exhibited large individual responses to obesogenic high-fat diet revealed distinct gender-specific mechanisms with respect to development of obesity and insulin resistance. Indeed, such gender-specific differences based on a complex metabolomics analysis have been also observed in human subjects in a recent study [65]. Our results reveal that several AC species as well as amino acids analyzed in fasting plasma samples could serve as a complex, gender-specific biomarker of propensity to obesity, and partially also as a biomarker of obesity-associated insulin resistance. Further studies are required to validate these potential biomarkers in human subjects.

## Supporting Information

S1 Data Data files underlying the figures. Detailed data tables are corresponding to the figures presented in the main body of the manuscript. Data tables corresponding to the S1 Fig are also included.
(XLSX)

S1 Fig Multivariate analysis of plasma metabolome. Plasma levels of AC species and amino acids in the fasting state were analyzed by the PLS-DA analysis in groups of mice selected for the highest and lowest ΔHOMA-IR. Score plots resulting from the PLS-DA analysis of plasma metabolite levels measured at week 4 (**A**, **C**) and week 22 (**B**, **D**) of the study were generated for both male (**A**, **B**) and female (**C**, **D**) mice. Plasma metabolites that were identified as those most discriminating between the groups of „high gainers”(empty dots) vs. „low gainers”(black dots) in both genders are listed in the tables under the respective score plots; the metabolites are ranked according to their variable influence on projection (VIP) scores, and only those with VIP scores greater than 1 are shown. Two animals due to missing values were excluded from further analysis.
(EPS)

S1 Table Plasma acylcarnitines and amino acids levels in week 4. Data are means ± SD. ^a^significantly different from Males STD; ^b^significantly different from Females STD; ^c^significantly different from Females HFD. Plasma aclycarnitine and amino acids levels are expressed in nmol/l.
(DOCX)

S2 Table Plasma acylcarnitines and amino acids levels in week 22. Data are means ± SD. ^a^significantly different from Males STD; ^b^significantly different from Females STD; ^c^significantly different from Females HFD. Plasma aclycarnitine and amino acids levels are expressed in nmol/l.
(DOCX)
