# Supplementary material for: Plasma Acylcarnitines and Amino Acid Levels As an Early Complex Biomarker of Propensity to High-Fat Diet-Induced Obesity in Mice
Source: PLoS One. 2016 May 16;11(5):e0155776. doi: 10.1371/journal.pone.0155776 (PMC4868278; doi:10.1371/journal.pone.0155776)
Supplement: S1 Table — Data are means ± SD. asignificantly different from Males STD; bsignificantly different from Females STD; csignificantly different from Females HFD. Plasma aclycarnitine and amino acids levels are expressed in nmol/l. (DOCX) [file pone.0155776.s003.docx]

| **Supplementary table 1. Plasma acylcarnitines and amino acids levels in week 4** | | | | | | | | | | | | | | | | |
| --- | --- | --- | --- | --- | --- | --- | --- | --- | --- | --- | --- | --- | --- | --- | --- | --- |
|  |  | **Male** | | | | | | |  | **Female** | | | | | | |
|  |  | **STD** | | |  | **HFD** | | |  | **STD** | | |  | **HFD** | | |
|  | |  |  |  |  |  |  |  |  |  |  |  |  |  |  |  |
| *Car* | | 2488 | ± | 1141 |  | 2707 | ± | 932^c^ |  | 3408 | ± | 1410 |  | 3103 | ± | 1140 |
| *C2* | | 2723 | ± | 1149 |  | 2914 | ± | 751 |  | 3164 | ± | 809 |  | 3241 | ± | 1087 |
| *C3* | | 132 | ± | 54 |  | 143 | ± | 62 |  | 156 | ± | 50 |  | 161 | ± | 72 |
| *C4* | | 168 | ± | 69 |  | 153 | ± | 57^a,c^ |  | 199 | ± | 70 |  | 181 | ± | 79 |
| *C5* | | 143 | ± | 68 |  | 135 | ± | 52 |  | 129 | ± | 36 |  | 129 | ± | 59 |
| *C3DC,C4OH* | | 38 | ± | 19 |  | 44 | ± | 16 |  | 35 | ± | 9 |  | 46 | ± | 25 |
| *C6* | | 44 | ± | 25 |  | 43 | ± | 15 |  | 42 | ± | 10 |  | 46 | ± | 21 |
| *C4DC,C5OH* | | 20 | ± | 8 |  | 21 | ± | 6 ^c^ |  | 25 | ± | 6 |  | 23 | ± | 7 |
| *C8* | | 13 | ± | 9 |  | 15 | ± | 9 |  | 28 | ± | 32 |  | 14 | ± | 9 |
| *C10-1* | | 16 | ± | 8 |  | 17 | ± | 5 |  | 18 | ± | 3 |  | 17 | ± | 5 |
| *C10* | | 9 | ± | 4 |  | 11 | ± | 5 |  | 14 | ± | 8 |  | 11 | ± | 5 |
| *C12* | | 24 | ± | 12 |  | 29 | ± | 14 |  | 26 | ± | 7 |  | 29 | ± | 20 |
| *C14-2* | | 12 | ± | 8 |  | 13 | ± | 5 ^c^ |  | 11 | ± | 3 |  | 12 | ± | 6 |
| *C14-1* | | 50 | ± | 28 |  | 55 | ± | 21 |  | 49 | ± | 8 |  | 53 | ± | 19 |
| *C14* | | 50 | ± | 27 |  | 62 | ± | 30 |  | 52 | ± | 12 |  | 58 | ± | 28 |
| *C16-1* | | 41 | ± | 25 |  | 45 | ± | 25 |  | 40 | ± | 12 |  | 43 | ± | 21 |
| *C16* | | 125 | ± | 70 |  | 152 | ± | 66 |  | 118 | ± | 31 |  | 141 | ± | 59 |
| *C16-1OH* | | 2 | ± | 1 |  | 3 | ± | 1 |  | 2 | ± | 1 |  | 3 | ± | 1 |
| *C16OH* | | 2 | ± | 1 |  | 3 | ± | 1 |  | 2 | ± | 0^a^ |  | 2 | ± | 1 |
| *C18-2* | | 79 | ± | 42 |  | 90 | ± | 38 ^c^ |  | 76 | ± | 20 |  | 79 | ± | 34 |
| *C18-1* | | 139 | ± | 68 |  | 162 | ± | 66 ^c^ |  | 128 | ± | 31 |  | 145 | ± | 58 |
| *C18* | | 34 | ± | 14 |  | 41 | ± | 11 ^c^ |  | 35 | ± | 8 |  | 36 | ± | 8 |
| *C18-1OH* | | 8 | ± | 3 |  | 9 | ± | 3 |  | 8 | ± | 2 |  | 9 | ± | 2 |
| *C18OH* | | 4 | ± | 2 |  | 5 | ± | 1 |  | 5 | ± | 1 |  | 5 | ± | 1 |
| *C16DC* | | 26 | ± | 11 |  | 25 | ± | 7 |  | 26 | ± | 7 |  | 25 | ± | 6 |
| *C20* | | 7 | ± | 2 |  | 8 | ± | 2 |  | 8 | ± | 1 |  | 8 | ± | 2 |
| *C20-4* | | 14 | ± | 7 |  | 16 | ± | 5 |  | 15 | ± | 3 |  | 15 | ± | 4 |
| *C2-C7* | | 3268 | ± | 1357 |  | 3451 | ± | 859 |  | 3750 | ± | 949 |  | 3829 | ± | 1232 |
| *C8-C14* | | 175 | ± | 86 |  | 202 | ± | 71 |  | 197 | ± | 55 |  | 194 | ± | 70 |
| *C16-C26* | | 482 | ± | 234 |  | 560 | ± | 207 |  | 463 | ± | 104 |  | 511 | ± | 179 |
| *All C2-C26* | | 3924 | ± | 1638 |  | 4213 | ± | 930 |  | 4411 | ± | 985 |  | 4534 | ± | 1270 |
| *Ala* | | 193210 | ± | 86253 |  | 195657 | ± | 85094 |  | 198535 | ± | 30613 |  | 223189 | ± | 79796 |
| *Pro* | | 137373 | ± | 53056 |  | 153454 | ± | 78390 |  | 138479 | ± | 28585 |  | 163727 | ± | 74470 |
| *Val* | | 643703 | ± | 326054 |  | 664736 | ± | 268863 |  | 590880 | ± | 284906 |  | 663807 | ± | 231105 |
| *Thr* | | 48394 | ± | 22920 |  | 52283 | ± | 28752 |  | 46216 | ± | 15388 |  | 55741 | ± | 24927 |
| *Leu+Ile* | | 137654 | ± | 70435 |  | 139428 | ± | 57695 |  | 124395 | ± | 56982 |  | 141107 | ± | 52046 |
| *Orn* | | 47841 | ± | 18806 |  | 58865 | ± | 32542 |  | 52540 | ± | 11621 |  | 63783 | ± | 29685 |
| *Lys* | | 12057 | ± | 4814 |  | 12388 | ± | 3831 |  | 12364 | ± | 1444 |  | 12516 | ± | 2887 |
| *Gln* | | 480270 | ± | 184741 |  | 504068 | ± | 144420 |  | 481824 | ± | 68438 |  | 524991 | ± | 108828 |
| *Arg* | | 194265 | ± | 76797 |  | 214615 | ± | 87660 ^c^ |  | 221522 | ± | 42547 |  | 250992 | ± | 77049 |
| *Tyr* | | 187563 | ± | 94756 |  | 201226 | ± | 92997 |  | 177050 | ± | 43723 |  | 217007 | ± | 98660 |
| *Trp* | | 440312 | ± | 190135 |  | 480120 | ± | 203609 |  | 492260 | ± | 120865 |  | 520890 | ± | 160976 |
| *BCAA* | | 781357 | ± | 395275 |  | 804164 | ± | 324564 |  | 715275 | ± | 341438 |  | 804914 | ± | 281361 |
| *ALL AA* | | 2522641 | ± | 1015177 |  | 2676840 | ± | 926810 |  | 2536065 | ± | 495893 |  | 2837749 | ± | 757247 |
|  | |  |  |  |  |  |  |  |  |  |  |  |  |  |  |  |
|  |  |  |  |  |  |  |  |  |  |  |  |  |  |  |  |  |
| Data are means ± SD. | | | | | | | | | | | | | | | | |
| ^a^significantly different from Males STD; ^b^significantly different from Females STD; ^c^significantly different from Females HFD. Plasma aclycarnitine and amino acids levels are expressed in nmol/l. | | | | | | | | | | | | | | | | |
