# Supplementary material for: Plasma Acylcarnitines and Amino Acid Levels As an Early Complex Biomarker of Propensity to High-Fat Diet-Induced Obesity in Mice
Source: PLoS One. 2016 May 16;11(5):e0155776. doi: 10.1371/journal.pone.0155776 (PMC4868278; doi:10.1371/journal.pone.0155776)
Supplement: S2 Table — Data are means ± SD. asignificantly different from Males STD; bsignificantly different from Females STD; csignificantly different from Females HFD. Plasma aclycarnitine and amino acids levels are expressed in nmol/l. (DOCX) [file pone.0155776.s004.docx]

| **Supplementary table 2. Plasma acylcarnitines and amino acids levels in week 22** | | | | | | | | | | | | | | | | |
| --- | --- | --- | --- | --- | --- | --- | --- | --- | --- | --- | --- | --- | --- | --- | --- | --- |
|  |  | **Male** | | | | | | |  | **Female** | | | | | | |
|  |  | **STD** | | |  | **HFD** | | |  | **STD** | | |  | **HFD** | | |
|  | |  |  |  |  |  |  |  |  |  |  |  |  |  |  |  |
| *Car* | | 8078 | ± | 5578 |  | 10409 | ± | 3246 ^a,c^ |  | 5871 | ± | 1248 |  | 11437 | ± | 3828^b^ |
| *C2* | | 22939 | ± | 5775 |  | 32347 | ± | 7015 ^a^ |  | 21674 | ± | 4428 |  | 32858 | ± | 6285 ^b^ |
| *C3* | | 374 | ± | 167 |  | 494 | ± | 148 ^a^ |  | 303 | ± | 113 |  | 473 | ± | 138 ^b^ |
| *C4* | | 536 | ± | 224 |  | 760 | ± | 178 ^a^ |  | 434 | ± | 116 |  | 761 | ± | 164 ^b^ |
| *C5* | | 418 | ± | 107 |  | 440 | ± | 123 |  | 338 | ± | 92^a^ |  | 453 | ± | 156 ^b^ |
| *C3DC,C4OH* | | 495 | ± | 161 |  | 1003 | ± | 349 ^a^ |  | 533 | ± | 144 |  | 1019 | ± | 312 ^b^ |
| *C6* | | 240 | ± | 81 |  | 238 | ± | 57 ^a,c^ |  | 188 | ± | 34^a^ |  | 219 | ± | 55 |
| *C4DC,C5OH* | | 69 | ± | 20 |  | 56 | ± | 16 ^a^ |  | 71 | ± | 13 |  | 59 | ± | 17 ^b^ |
| *C8* | | 46 | ± | 17 |  | 53 | ± | 14 |  | 48 | ± | 13 |  | 52 | ± | 15 |
| *C10-1* | | 60 | ± | 13 |  | 87 | ± | 22 ^a,c^ |  | 52 | ± | 9^a^ |  | 82 | ± | 21 ^b^ |
| *C10* | | 44 | ± | 10 |  | 45 | ± | 11 ^a^ |  | 41 | ± | 6 |  | 43 | ± | 12 |
| *C12* | | 90 | ± | 28 |  | 78 | ± | 19 ^a^ |  | 79 | ± | 18 |  | 82 | ± | 22 |
| *C14-2* | | 78 | ± | 24 |  | 91 | ± | 26 |  | 64 | ± | 14^a^ |  | 86 | ± | 22 ^b^ |
| *C14-1* | | 281 | ± | 92 |  | 214 | ± | 53 ^a,c^ |  | 275 | ± | 50 |  | 186 | ± | 42 ^b^ |
| *C14* | | 234 | ± | 88 |  | 175 | ± | 48 ^a^ |  | 218 | ± | 34 |  | 168 | ± | 44 ^b^ |
| *C16-1* | | 212 | ± | 89 |  | 121 | ± | 44 ^a,c^ |  | 209 | ± | 26 |  | 99 | ± | 34 ^b^ |
| *C16* | | 466 | ± | 141 |  | 354 | ± | 80 ^a^ |  | 446 | ± | 60 |  | 338 | ± | 69 ^b^ |
| *C16-1OH* | | 12 | ± | 4 |  | 15 | ± | 4 |  | 11 | ± | 2a |  | 14 | ± | 3 ^b^ |
| *C16OH* | | 22 | ± | 7 |  | 20 | ± | 6 ^a^ |  | 22 | ± | 4 |  | 19 | ± | 6 ^b^ |
| *C18-2* | | 305 | ± | 58 |  | 513 | ± | 163 ^a,c^ |  | 257 | ± | 43^a^ |  | 440 | ± | 104 ^b^ |
| *C18-1* | | 706 | ± | 188 |  | 772 | ± | 213^c^ |  | 830 | ± | 121^a^ |  | 681 | ± | 153 ^b^ |
| *C18* | | 105 | ± | 30 |  | 149 | ± | 33 ^a^ |  | 124 | ± | 13^a^ |  | 145 | ± | 28 |
| *C18-1OH* | | 39 | ± | 18 |  | 45 | ± | 19 |  | 52 | ± | 8^a^ |  | 43 | ± | 17 ^b^ |
| *C18OH* | | 12 | ± | 4 |  | 17 | ± | 5 ^a^ |  | 17 | ± | 4^a^ |  | 15 | ± | 5 ^b^ |
| *C16DC* | | 56 | ± | 35 |  | 98 | ± | 35 ^a,c^ |  | 68 | ± | 13^a^ |  | 131 | ± | 37 ^b^ |
| *C20* | | 8 | ± | 3 |  | 6 | ± | 3 ^a^ |  | 12 | ± | 4^a^ |  | 6 | ± | 3 ^b^ |
| *C20-4* | | 35 | ± | 10 |  | 46 | ± | 14 |  | 48 | ± | 10^a^ |  | 47 | ± | 12 ^b^ |
| *C2-C7* | | 25070 | ± | 6203 |  | 35339 | ± | 7543 ^a^ |  | 23541 | ± | 4841 |  | 35842 | ± | 6828 ^b^ |
| *C8-C14* | | 833 | ± | 233 |  | 743 | ± | 161 ^a^ |  | 777 | ± | 127 |  | 698 | ± | 153 ^b^ |
| *C16-C26* | | 1980 | ± | 480 |  | 2156 | ± | 526^c^ |  | 2097 | ± | 267 |  | 1978 | ± | 379 ^b^ |
| *All C2-C26* | | 27883 | ± | 6179 |  | 38238 | ± | 7871 ^a^ |  | 26415 | ± | 5097 |  | 38519 | ± | 7058 ^b^ |
| *Ala* | | 234437 | ± | 87734 |  | 245923 | ± | 53833 |  | 216966 | ± | 21733 |  | 244605 | ± | 67243 |
| *Pro* | | 289122 | ± | 35773 |  | 251634 | ± | 49863 ^a^ |  | 297009 | ± | 62146 |  | 249915 | ± | 49330 ^b^ |
| *Val* | | 231432 | ± | 60052 |  | 283024 | ± | 60090 |  | 259433 | ± | 35739 |  | 287053 | ± | 63392 |
| *Thr* | | 2309636 | ± | 659861 |  | 2734635 | ± | 646946 |  | 2153328 | ± | 243580 |  | 2750600 | ± | 913399 |
| *Leu+Ile* | | 257919 | ± | 30792 |  | 225596 | ± | 50069 ^a^ |  | 276744 | ± | 55770 |  | 224543 | ± | 45539 ^b^ |
| *Orn* | | 183720 | ± | 48212 |  | 234423 | ± | 85652^c^ |  | 181861 | ± | 45689 |  | 261964 | ± | 108146 ^b^ |
| *Lys* | | 18936 | ± | 5481 |  | 24518 | ± | 7183 ^a,c^ |  | 22268 | ± | 4283 |  | 27174 | ± | 8164 |
| *Gln* | | 698172 | ± | 231528 |  | 786987 | ± | 222700^c^ |  | 865706 | ± | 128444^a^ |  | 886068 | ± | 260943 |
| *Arg* | | 630920 | ± | 121598 |  | 781750 | ± | 244308 |  | 663372 | ± | 138908 |  | 800183 | ± | 269533 |
| *Tyr* | | 592670 | ± | 217036 |  | 787114 | ± | 253997 ^a,c^ |  | 438779 | ± | 166655^a^ |  | 685552 | ± | 203748 ^b^ |
| *Trp* | | 610332 | ± | 181116 |  | 924665 | ± | 219447 ^a^ |  | 565115 | ± | 131795 |  | 956295 | ± | 219058 ^b^ |
| *BCAA* | | 489351 | ± | 68103 |  | 508620 | ± | 97994 ^a^ |  | 536176 | ± | 81339 |  | 511596 | ± | 97205 ^b^ |
| *ALL AA* | | 6057298 | ± | 1324096 |  | 7280268 | ± | 1514008 |  | 5940580 | ± | 725741 |  | 7373953 | ± | 1785522 |
|  | |  |  |  |  |  |  |  |  |  |  |  |  |  |  |  |
|  | |  |  |  |  |  |  |  |  |  |  |  |  |  |  |  |
| Data are means ± SD. | | | | | | | | | | | | | | | |  |
| ^a^significantly different from Males STD; ^b^significantly different from Females STD; ^c^significantly different from Females HFD. Plasma aclycarnitine and amino acids levels are expressed in nmol/l. | | | | | | | | | | | | | | | | |
